# Phase Transitions and Stabilities among Three Phases of Di-p-tolyl Disulfides

**DOI:** 10.3390/molecules27238342

**Published:** 2022-11-30

**Authors:** Imran Ali, Yanqiang Han, Jinjin Li

**Affiliations:** Key Laboratory for Thin Film and Microfabrication of Ministry of Education, Department of Micro/Nano Electronics, School of Electronic Information and Electrical Engineering, Shanghai Jiao Tong University, Shanghai 200240, China

**Keywords:** disulfides, di-p-tolyl disulfides (p-Tol_2_S_2_), polymorphs, high pressures, lattice parameters, Gibbs free energy, temperature induction, phase transformation, vibrational spectroscopy, FT–IR spectroscopy

## Abstract

Di-p-tolyl disulfides (p-Tol_2_S_2_) are employed as load-carrying additives because of their anti-wear and extreme load-bearing qualities. External pressure triggers conformational up-conversion (leads to phase transition) in the molecules of p-Tol_2_S_2_, by compensating for the stress and absorbing its energy. These features make p-Tol_2_S_2_ a potential candidate for next-generation energy storage devices. Upon lithiation, MoS_2_ expands up to 103% which causes stress and affects battery stability and performance. Therefore, it is essential to study these materials under different physical conditions. In this work, we used density functional theory (DFT) at *ω*B97XD/6-31G* functional level, to calculate lattice parameters, Gibbs free energies, and vibrational spectra of three phases (i.e., α, β, and γ) of p-Tol_2_S_2_ under different pressure and temperature conditions. The phase transition between phases α and β occurred at a pressure and temperature of 0.65 GPa and 463 K, respectively. Furthermore, phase transition between phases α and γ was found at a pressure and temperature of 0.35 GPa and 400 K, respectively. Moreover, no phase transition was observed between phases β and γ under the pressure range studied (0 GPa to 5.5 GPa). We also computed and compared the FT–IR spectra of the three phases. These results can guide scientists and chemists in designing more stable battery materials.

## 1. Introduction

Sulfides have numerous applications in the preparation of lubricants [1], agricultural pesticides [2], medicines [2,3], energy storage devices [4,5,6,7,8], photovoltaics [9,10,11,12], etc. For more than half a century, organic sulfides have been significantly employed as load-carrying additives in lubricants [1], because of their anti-wear and extreme load-bearing qualities [1,2]. In addition, most organic compounds containing the sulfide group are used in agriculture for the preparation of pesticides [2]. Furthermore, sulfide groups with high lipophilicity are potentially important for pharmaceutical industries because they can increase the solubility of medicine in lipids [2,3]. Moreover, in the 1970s pioneers started utilizing metal sulfides in lithium batteries [13]. For lithium–sulfur batteries, sulfur is considered an ideal cathode candidate for the next generation energy storage devices owing to fact that the element sulfur is an abundant, non-toxic and inexpensive cathode material that provides higher theoretical energy density and specific capacity than commercially available lithium-ion batteries [14,15]. Metal sulfides have various applications in photovoltaics which convert solar energy directly into electrical energy. Metal sulfides, such as CdS [16,17], Ag_2_S [18], CuInS_2_ [19], and PbS [20,21], are used for harvesting solar energy. Disulfides can significantly increase bearing properties under extreme loads; therefore, they have been used for decades as additives in lubricants [1]. Furthermore, p-Tol_2_S_2_ is also used as an electrolyte additive in batteries to improve high voltage performance [22,23]. It was demonstrated that organic disulfides performed better under high loads as they progressed from phenyl, across n-butyl, s-butyl, and t-butyl to benzyl [24]. A further indication that conformational changes, and the strength of the S−S bonds, have a role in load-bearing qualities is the fact that organic disulfides’ anti-wear properties arise from n-butyl, allyl, and benzyl to phenyl [25]. These microscopic and macroscopic properties of organic disulfides lead to numerous technical applications. Diphenyl-disulfide exists in 182 different polymorphic forms, deposited in the Cambridge Structural Database after X-ray diffraction observations [25], out of which 122 are crystals with a single independent molecule (Z′ = 1), 44 are structures in the asymmetric unit cell (Z′ = 0.5), 10 are crystals with Z′ = 2, 5 are crystals with 1.5 independent molecules (Z′ = 1.5), and 1 structure has Z′ = 4 [25]. The application of high pressure was used to develop new polymorphs of several compounds. such as active pharmaceutical ingredients [26,27,28,29,30,31,32], relaxors [33,34], semiconductors [35], etc. High pressures efficiently reduce the volume of organic compounds and could modify molecular conformations and intermolecular interactions. Silicon is considered a potential anode material for Lithium-ion batteries because of its high theoretical capacity, which is roughly tenfold higher than commercially available graphite anodes [36,37]. However, silicon is not commercially utilized for lithium-ion batteries because of its expansion of up to 300% [38]. Ali et al. studied stress evolution in silicon-based anode material caused by volume expansion over lithiation [39]. Disulfide (MoS_2_) expands up to 103% upon lithiation, as compared to silicon, which expands up to 280% upon full lithiation [40]. Organic disulfides can bear high stresses and keep on changing forms over increments of certain stresses without deformation [24]. Therefore, disulfides can be used in batteries to increase stability and battery life.

In this work, the phase transitions among the three phases of p-Tol_2_S_2_ were found, using a computational method. In order to determine the Gibbs free energy in the temperature range from 0 K to 500 K, with a step size of 1 K, ab initio calculations were carried out at various pressures. The cutting-edge equipment described here allowed for ab initio calculations of the Gibbs free energy of generic pharmaceutical crystals at finite temperatures and pressures, which assisted in the phase transition discovery process. To optimize crystal structures, we applied density functional theory (DFT) at the level of *ω*B97XD/6-31G* functionality. Since organic compounds typically have enormous molecular sizes, the conventional ab initio computational technique does not work with them. Therefore, we used the embedded fragmentation quantum mechanical approach [41,42,43,44]. It is a technique that divides the internal energy of a p-Tol_2_S_2_ crystal into appropriate mixtures of monomers and overlapping dimer energies that are incorporated in the electrostatic field of the crystalline environment [44]. The Hartree–Fock level embedding field, which consists of self-consistently determined atomic charges, is a crucial technique. Large molecules and crystals can be successfully treated using the embedded fragment quantum mechanical approach since it includes one-body and two-body interactions. Additionally, the interaction energies between two fragments distanced by a threshold distance were calculated using quantum mechanics, and the interaction energies between long-range interacting fragments were calculated using charge–charge Coulomb interactions. The volumes, stabilities, Gibbs free energies, and FT–IR spectra of three phases of p-Tol_2_S_2_ were provided corresponding to different pressures. The computations of Gibbs free energies were successfully utilized to find phase transformation among different phases of polymorphic materials [45,46,47].

With the calculation of lattice parameters, Gibbs free energies, and vibrational spectra of three phases (i.e., α, β, and γ) of p-Tol_2_S_2_ under different pressure and temperature conditions, the phase transition between phases α and β was determined at pressure and temperature of 0.65 GPa and 463 K, respectively. Under the pressure of 0.65 GPa, phase α remained stable at a temperature lower than 463 K. However, at a temperature from 463 K to 500 K, phase β remained more stable. Moreover, no phase transition was observed between phases β and γ under the pressure range studied (0 GPa to 5.5 GPa). We also presented and compared FT–IR spectra of three phases of p-Tol_2_S_2_. This work can guide scientists to identify stable phases of polymorphic crystals under extreme conditions.

## 2. Results and Discussion

### 2.1. Crystal Structure Prediction

**Figure 1:**
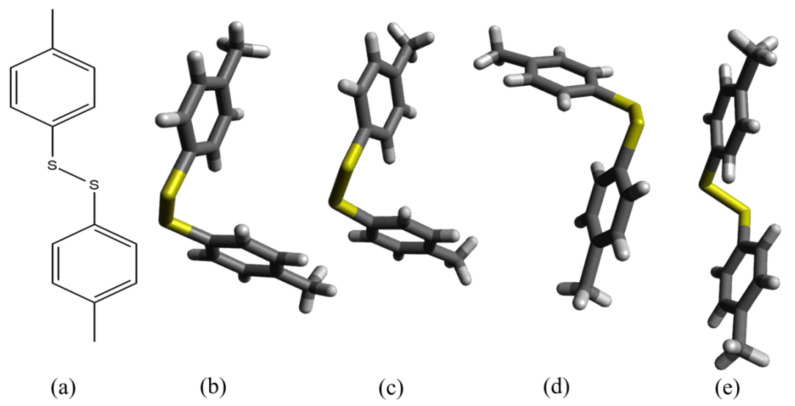
The structures of p-Tol_2_S_2_. (**a**) Shows molecular structure of p-Tol_2_S_2_. (**b**,**e**) show 3D structures (stick models) of phases α and γ, respectively. (**c**,**d**) show two conformations (stick models) of phase β.

The crystal structures of p-Tol_2_S_2_ phases α, β, and γ were taken from the Cambridge Structural Database with CSD refcodes of IPIXUB06, IPIXUB14, and IPIXUB08, respectively [25]. The phase α has a space group of P2_1_ and possesses a monoclinic unit cell with a volume of 636.417 Å^3^. Phase β has a space group of P1 that possesses a triclinic unit cell with a volume of 556.886 Å^3^. The phase γ has a space group of P2_1_/c that possesses a monoclinic unit cell with a volume of 1199.544 Å^3^. The crystal structure parameters of phases α, β, and γ were collected at atmospheric pressure, 2.2 GPa, and 0.45 GPa, respectively, whereas ambient temperature was used for all data collection [25]. The molecular structure and 3D structures in stick models of p-Tol_2_S_2_ are given in Figure 1. The comparison of observed [25] and computed lattice parameters of three phases of p-Tol_2_S_2_ are given in Table 1, where all angles were kept fixed. The crystal structure parameters were computed at temperature of 0 K. The computed structure parameters were rather different from the experimental [25]; however, the minimum, maximum and average differences were about 0.039, 0.245, and 0.187, respectively, which was acceptable. In addition, lattice parameters of all three phases calculated under high pressures are given in Table A1. Almost all of the lattice parameters followed the same trend of reduction in length under application of increasing pressure; for details see Table A1.

### 2.2. Pressure Dependence Volume

**Figure 2:**
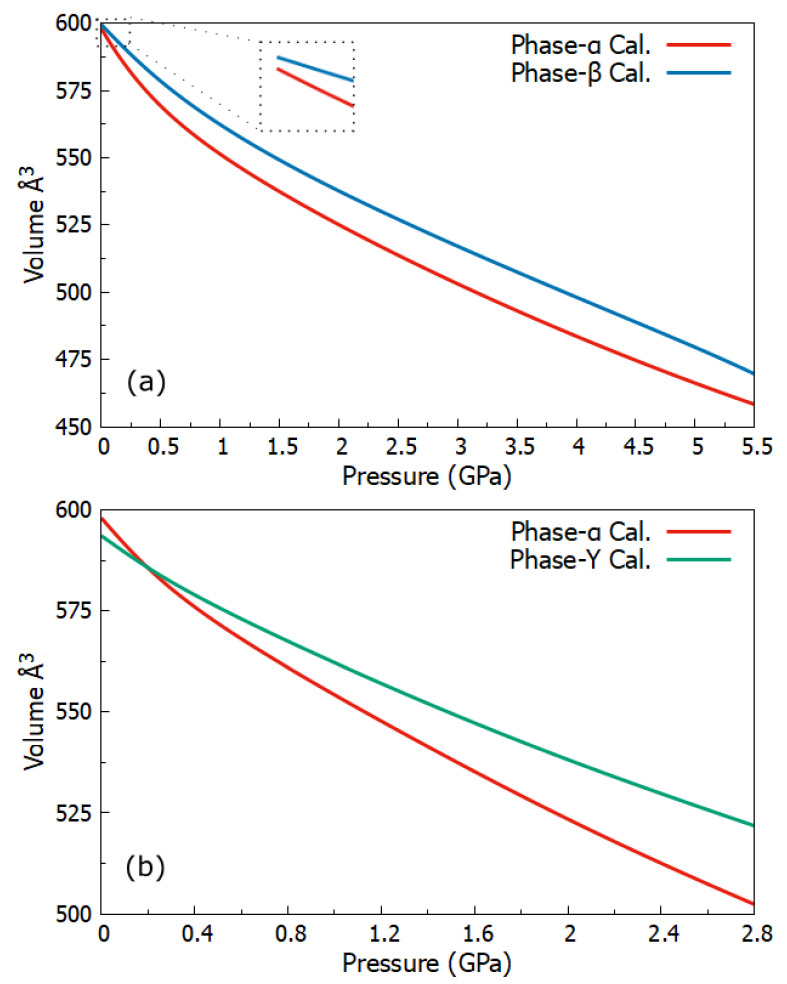
The ab initio calculated change in volume for three phases of p-Tol_2_S_2_ with respect to pressure, all curves were fitted with the Bezier function. (**a**) Shows volume change in phases α and β by increasing pressure from atmospheric to 5.5 GPa. (**b**) Shows volume change in phases α and γ by increasing pressure from atmospheric to 2.8 GPa.

We calculated the crystal structure parameters of three phases of p-Tol_2_S_2_ with variable pressures. The crystal structure parameters were optimized under high pressures from 0 GPa to 5.5 GPa. For pressures, we did not use a specific step size. Initially, we used the same pressure values as experimental work done before by Sobczak and Katrusiak [25], so that we could validate our computational results. The most important property to study for a crystal under pressure was the volume change. In addition, the pressure and volume relationship could confirm if phase transition occurred, due to the change in volume caused by application of pressure. Figure 2 shows the change in volume corresponding to pressure for three phases of p-Tol_2_S_2_.

We can observe that as pressure increased, the volumes of the two phases α and β decreased almost similarly, as shown in Figure 2a. No abrupt change in volumes of the two phases was observed. Therefore, phase transition between phases α and β was not directly related to the change in volumes corresponding to pressure. In contrast, the phase transition between α and γ was observed to be related to volume change due to pressure. From Figure 2b we can see the sudden drop in the volume of phase α as pressure was raised from 0 GPa to 0.35 GPa. This drop was likely due to compression of the short intermolecular contacts, which led to phase transition between phases α and γ. These results were also consistent with the experimental results presented by Sobczak and Katrusiak [25]. Next, we present the effect of high pressure on Gibbs free energies of three phases of p-Tol_2_S_2_.

### 2.3. Gibbs Free Energy Difference

**Figure 3:**
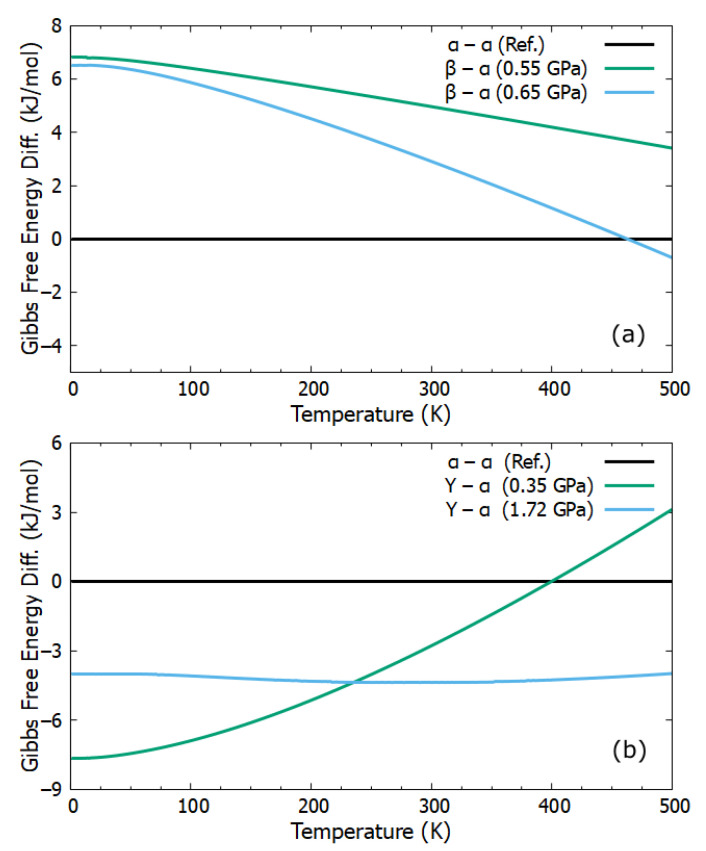
The calculated Gibbs free energy differences concerning temperature and different pressures. (**a**) Shows Gibbs free energy differences between phases α and β. (**b**) Shows Gibbs free energy differences between phases α and γ.

**Figure 4:**
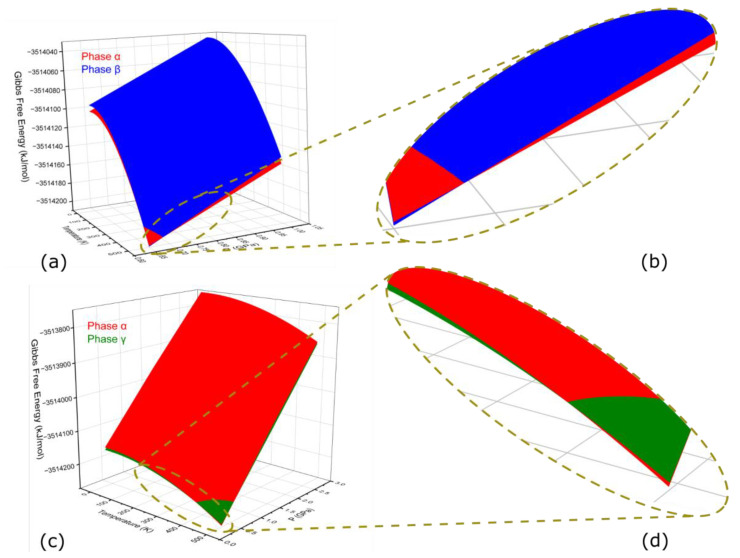
The effects of pressures and temperatures on Gibbs free energies (GFEs). Sub-figure (**a**,**c**) show a 3D surface plot of phases α, β, and α, γ, respectively. Sub-figure (**b**,**d**) show magnified portions of sub-figure (**a**,**b**), respectively. The red, blue, and green colors represent the phases α, β, and γ, respectively.

Gibbs free energy (GFE) was successfully used to study stability and phase transition between/among different phases of polymorphs [46,48,49,50]. Here we provide the change in GFE differences concerning temperature, see Figure 3. The GFE differences of phases α, β and α, γ are given in Figure 3a,b, respectively. The phase α remained stable at a fixed pressure and a temperature range of 0.55 GPa and 0 K–500 K, respectively. The phase transition between phases α and β was observed at a pressure and temperature of 0.65 GPa and 463 K, respectively. Under the pressure of 0.65 GPa, phase α remained stable at a temperature lower than 463 K. However, at a temperature from 463 K to 500 K phase β remained more stable. The phase transition between phases α and γ was observed at a pressure and temperature of 0.35 GPa and 400 K, respectively. In addition, we also generated a 3D plot to observe the effects of temperature and pressure on GFEs, see Figure 4.

The GFEs increased negatively from top to bottom, so, therefore, the surface on the bottom side was more stable. In Figure 4a, the blue surface represents the area where phase β was less stable than phase α. However, the red surface on the bottom left represents the area where phase β was more stable than phase α. In other words, the phase β was only stable at a pressure from 0.65 GPa to 0.7 GPa and temperature from 460 K to 500 K, see Figure 4a,b. Whereas, phase α was stable in the remaining whole surface studied. Figure 4c,d can be explained similarly to Figure 4a,b, respectively. The phase γ was more stable than phase α at high-pressure and/or low-temperature conditions. From Figure 4a,b, we can see a general trend of an increase in GFEs concerning an increase in pressure. Furthermore, a decrease in GFEs was observed as the temperature was increased. Therefore, same phase transitions could be determined from the GFEs. as demonstrated in Figure 3.

### 2.4. Vibrational Spectra

The molecular structure, the type of chemical bond, and the intramolecular forces acting between the atoms in a molecule are all essential topics covered by vibrational spectroscopy [51]. It is a potent tool for the physical evaluation of pharmaceutical solids, especially when performed using the Fourier transform method (FT–IR or FT–Raman) [52]. In one of our previous articles, Raman spectra of p-Tol_2_S_2_ phases α and β were covered in detail [44]. Therefore, only IR spectra are provided here.

#### FT–IR Spectra

FT–IR spectra are frequently employed to evaluate the type of polymorphism present in a pharmaceutical substance [53]. The DFT computational methods have been successfully used to predict FT–IR spectra of different pharmaceutical crystals [44,48,50]. Here, for comparison, we provide FT–IR of three phases of p-Tol_2_S_2_.

**Figure 5:**
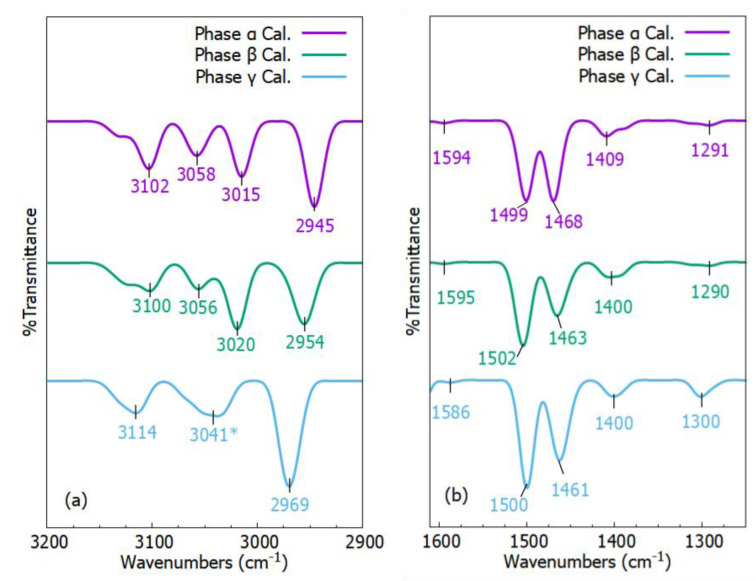
The calculated FT−IR spectra (at atmospheric pressure) of phases α, β, and γ, which are represented by purple, green, and blue colored curves, ranging from (**a**) 3200 cm^−1^ to 2900 cm^−1^ and (**b**) 1600 cm^−1^ to 1250 cm^−1^, respectively.

**Figure A1:**
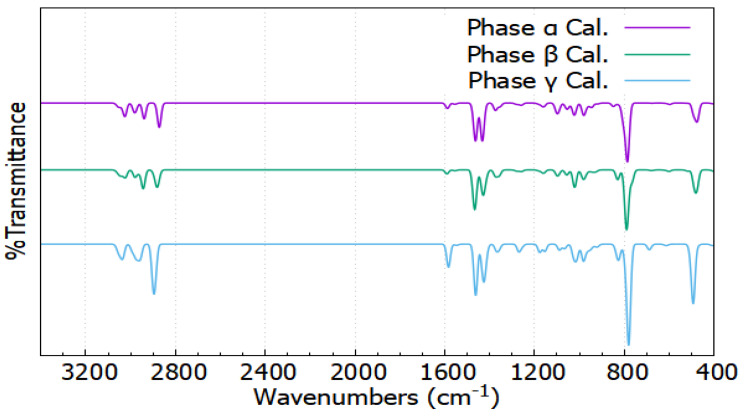
The comparison of calculated FT−IR spectra of α, β and γ phases which are represented by purple, green and blue colored curves, respectively.

FT–IR spectra of three phases of p-Tol_2_S_2_ are given in Figure 5. Here, we selected two portions of FT–IR spectra for comparison. The full spectra can be seen in Figure A1. Figure 5a,b represent FT–IR spectra, ranging from 3200 cm^−1^ to 2900 cm^−1^ and 1600 cm^−1^ to 1250 cm^−1^, respectively. The main difference among the three phases can be seen at wavenumbers from 3015 cm^−1^ to 3114 cm^−1^ in Figure 5a. In this range phases α and β had three absorption peaks while phase γ had only two absorption peaks. Another main difference was the absorption peak shape at around 3100 cm^−1^. Phase α showed a sharp peak at 3102 cm^−1^ with a shoulder peak, whereas phases β and γ showed a broad peak (at 3100 cm^−1^) with a shoulder peak and a broad peak without a shoulder peak (at 3114 cm^−1^), respectively. The change of sharp peak to a broad peak was related to overlapping of peaks. In Figure 5b, a similar trend can be observed at wavenumbers of 1400 cm^−1^, 1400 cm^−1^, and 1409 cm^−1^ for phases α, β, and γ, respectively. Furthermore, we assigned modes to most of the characteristic peaks of the three phases, given in Table 2.

From the literature, four normal modes were seen at high frequencies of 3083 cm^−1^, 3052 cm^−1^, 3043 cm^−1^, and 2943 cm^−1^. All of the modes were contributed to by C–H stretching vibrations [54]. Their corresponding calculated absorption frequencies of phases α, β, and γ are provided in Table 2. There is a large frequency difference between absorption peaks of 2943 cm^−1^, and 1596 cm^−1^, which is because of the high force constant for stretching vibrations [54]. Furthermore, energy for stretching vibration is higher than torsion and bending energy, therefore most stretching vibration modes occur at high frequencies.

The bending of the H atom on the benzene ring corresponds to modes observed at 1494 cm^−1^, 1452 cm^−1^, 1139 cm^−1^, and 1068 cm^−1^ taken from experimental work [54]. The corresponding frequencies and modes for phases α, β, and γ are given in Table 2. The absorption peak at 1181 cm^−1^ had very strong coupling characteristics. Except for disulfide bonds, all atoms were engaged, and it appeared that both hydrogen atom bending and apparent stretching of the carbon skeleton happened. FT–IR peak absorption at 1028 cm^−1^ was contributing to the bending and stretching of the carbon skeleton. The absorption peaks at 1001 cm^−1^, 803 cm^−1^, and 617 cm^−1^ corresponded to the carbon skeleton bending vibration in the benzene ring. At absorption peaks of 971 cm^−1^ and 695 cm^−1^, there was out-of-plane bending of hydrogen atoms on the benzene ring. Multiple vibrations were observed at 658 cm^−1^, especially, C–S stretching, and in-plane, and out-of-plane bending of the benzene ring.

## 3. Methods

The Quasi-Newton algorithm was implemented to optimize crystal structures [57]. As the molecular size of disulfides was large, we used an embedded-fragment method, with a DFT level of *ω*B97XD/6-31G*, to compute the Gibbs free energy of three phases of p-Tol_2_S_2_ [58]. The BFGS procedure [59] was used to update the approximation of the Hessian matrix and the maximum gradient was set to 0.001 Hartree/Bohr for convergence. The embedded fragment method was explained in detail in [48]. To treat the macromolecules effectively, the total energy of a unit cell of the crystal was divided into combinations of energies of monomers and dimers by the embedded fragment method. Each molecule was considered as a segment, and the energies between two segments which were close to each other were computed by quantum mechanics. The interaction energies between segments which were away from each other were calculated by interaction of coulomb’s charges. The calculation of the internal energy of a unit cell for a crystal system is given as:

(1) $$E_{i}=\underset{x}{\sum }E_{x\left(0\right)}+\underset{\begin{array}{c} x,y,x<y\\ R_{xy}\leq \lambda \end{array}}{\sum }\left(E_{x\left(0\right)y\left(0\right)}-E_{x\left(0\right)}-E_{y\left(0\right)}\right)\;+\frac{1}{2}\overset{S}{\underset{n=-S}{\sum }}\left(1-\delta_{n0}\right)\underset{\begin{array}{c} x,y\\ R_{xy}\leq \lambda \end{array}}{\sum }\left(E_{x\left(0\right)y\left(n\right)}-E_{i\left(0\right)}-E_{y\left(n\right)}\right)+T_{\mathrm{LR}}$$

where the three-integer index of the unit cell was represented by a variable ‘*n*’. The quantum mechanical energy of the *x*-th molecules in the *n*-th unit cell was represented by $E_{x\left(n\right)}$. The quantum mechanical energy of dimers was given by $E_{x\left(0\right)y\left(n\right)}$, where *x*-th and *y*-th denote the molecules’ number with respect to the *0*th central unit cell and *n*th unit cells [49,60,61]. The crystal system was defined by a 3×3×3 supercell. The $E_{x\left(0\right)}$ denoted the single molecular energy in the *0*-th unit cell, which was in the center, see Equation (1). The 2nd part of Equation (1) denoted the two-body quantum mechanical interaction that had a shorter distance than λ (where λ was a given cutoff distance which was set to 4 Å). The 3rd part of Equation (1) provided the interactions between one molecule in the central unit cell and the other in the *n*-th unit cell, which had a distance shorter than λ. Where *S* = 1, was the unit cell’s index. Quantum mechanics was used to compute short-range interactions (the first three parts in Equation (1)). It was calculated in the electrostatic field of the rest, where *ω*B97XD/6-31G* level was used to fit electrostatic potential variations. The background charges were represented by the 11×11×11 supercell. Coulomb’s interaction of charges was employed to approximately treat long-range interactions between two molecules of dimers with distance larger than λ. The long-range electrostatic interactions were denoted by $T_{\mathrm{LR}}$ in a 41×41×41 supercell. The enthalpy $E_{th}$ for each unit cell was computed by considering the effect of external pressure as follows:

(2) $$E_{th}=E_{i}+PV$$

where *P* and *V* denoted the external pressure and the unit cell volume, respectively.

(3) $$U_{v}=\frac{1}{K}\sum_{n}\sum_{k}\omega_{nk}\left(\frac{1}{2}+\frac{1}{e^{\beta \omega_{nk}}-1}\right)$$

(4) $$S_{v}=\frac{1}{\beta TK}\sum_{n}\sum_{k}\left\{\frac{\beta \omega_{nk}}{e^{\beta \omega_{nk}}-1}-\ln\left(1-e^{-\beta \omega_{nk}}\right)\right\}$$

(5) $$E_{gf}=E_{th}+U_{v}-TS_{v}$$

The harmonic approximation was utilized to compute zero-point vibrational energy $U_{v}$ and the entropy $S_{v}$, which were shown in Equations (3) and (4), respectively, where the phonon’s frequency with lattice vector *k* was represented by $\omega_{nk}$. $\beta =1/k_{0}T$ and $k_{0}$ was the Boltzmann constant. The capital *K* was the product of all *k*, which were evenly spaced grid points in the reciprocal unit cell. The *k*-grid of 21 × 21 × 21 was used in this study, where *K* = 9261. The calculation of Gibbs free energy (*E_gf_*) with effects of temperature and pressure in a unit cell was given by Equation (5). We employed DFT/*ω*B97XD/6-31G* to optimize lattice parameters, as well as to calculate enthalpy (*H_th_*), over single point energy, zero-point vibrational energy ($U_{v}$) and entropy ($S_{v}$).

After calculating the Gibbs free energy, the k-dependent force constant matrix, $F\left(k\right)$, could be calculated with the force constant matrix $\left(F_{n}\right)$ from the *0*th to the *i*th unit cells:

(6) $$F\left(k\right)=\overset{N}{\underset{0}{\sum }}F_{n}e^{inka},$$

where *a* denotes the translational period. Then the Raman spectra could be calculated by the following equation:

(7) $$R_{k}\propto \frac{3}{2}{\left(\sum_{p}^{a,b,c}\frac{\partial P_{ii}}{\partial Q_{k}}\right)}^{2}+\frac{21}{2}\sum_{i}^{a,b,c}\sum_{j}^{a,b,c}{\left(\frac{\partial P_{ij}}{\partial Q_{k}}\right)}^{2},$$

where *Q_k_* is the corresponding normal mode, and $\partial P_{ij}/\partial Q_{k}$ is the polarizability derivative. For Raman spectra, only the vibrations in a zero-center (**k** = 0) have nonzero intensities and are Raman-active. Thus, only the *0*th force constant matrix, $F\left(0\right)$, of the central cell (0, 0, 0) will be used for calculating the Raman frequency and intensity.

## 4. Conclusions

Disulfides are very famous for their high load-bearing qualities. Recently, their successful use in battery materials increased the research interest of scientists and chemists. Here we used the DFT computational method and calculated phase transition among three phases of p-Tol_2_S_2_. The phase transitions between phases α and β were determined at a pressure and temperature of 0.65 GPa and 463 K, respectively. Furthermore, phase transition between phases α and γ was found at a pressure and temperature of 0.35 GPa and 400 K, respectively. Moreover, no phase transition was observed between phases β and γ under the pressure range studied (0 GPa to 5.5 GPa). Furthermore, we also provided IR spectra and their assignments for three phases. Our computational results (i.e., crystal structure parameters, phase transitions, and IR peaks) were consistent with previous experimental work. This work not only accelerates the investigation of stability and phase transition of p-Tol_2_S_2_ under high-pressure conditions, but also provides guidelines for the design and development of new strong and stable battery materials.

## Figures and Tables

**Table 1:** The experimental [25] and computed crystal structure parameters of *α*, *β*, and *γ* phases of p-Tol_2_S_2_ at atmospheric pressure.

Parameters	a (Å)	b (Å)	c (Å)	*α* (deg)	*β* (deg)	*γ* (deg)	V (Å^3^)
Exp [25]. Phase *α*	7.593	5.713	14.722	90.00	94.76	90.00	636.417
Cal. Phase *α*	7.348	5.633	14.498	90.00	94.76	90.00	598.021
Exp [25]. Phase *β*	7.306	5.509	14.038	95.14	97.23	85.36	556.886
Cal. Phase *β*	7.541	5.662	14.245	95.14	97.23	85.36	599.465
Exp [25]. Phase *γ*	15.260	5.962	14.615	90.00	115.56	90.00	1199.544
Cal. Phase *γ*	15.155	5.923	14.661	90.00	115.56	90.00	1187.223

**Table 2:** Experimental and calculated IR spectra wavenumbers (cm^−1^) with mode assignments, where, υ: stretching, δ: Bending, δ_o_: out-of-plane bending, δ_i_: in-plane bending, N/A: not available.

Exp.	Cal. *α*	Cal. *β*	Cal. *γ*	Assignment	References
617	612	614	N/A	δ C-C	[54]
658	N/A	N/A	626	δ_o_/δ_i_ C—H, υ C—S / S—S	[54,55]
695	695	695	705	δ_o_ C—H	[54]
971	968	N/A	974	δ_o_ C—H	[54]
803	806	809	800	δ C—C	[54]
1001	1006	1006	1006	δ C—C	[54]
1028	1048	1047	1044	υ C—C, δ C—C	[54]
1068	1081	1083	1092	δ C—H	[54]
1139	1126	1126	1117	δ C—H	[54]
1181	1188	1188	1182	υ C—H, δ C—H	[54]
1291	1291	1290	1300	υ C—C	[54]
1452	1468	1463	1461	δ C—H	[54]
1494	1499	1502	1500	δ C—H	[54]
1596/1599	1594	1595	1588	υ C=C, υ C—C	[54,55]
2943	2945	2954	2969	υ C—H	[56]
3043	3015	3020	3041	υ C—H	[54]
3052	3058	3056	N/A	υ C—H	[54]
3083	3102	3100	3114	υ C—H	[54]

## Data Availability

The data presented in this study are available on request from the corresponding author.

## Appendix A

**Table A1:** Experimental and calculated crystal structure parameters of three phases of p-Tol_2_S_2_.

Phase	P (GPa)	a (Å)	b (Å)	c (Å)	*α* (deg)	*β* (deg)	*γ* (deg)	V (Å^3^)
α	Exp. atm	7.593	5.713	14.722	90.00	94.76	90.00	636.417
	Cal. atm	7.348	5.633	14.498	90.00	94.76	90.00	598.021
	0.25	7.212	5.59	14.411	90.00	94.76	90.00	578.977
	0.45	7.164	5.566	14.36	90.00	94.76	90.00	570.628
	0.65	7.081	5.565	14.253	90.00	94.76	90.00	559.713
	1	6.999	5.540	14.170	90.00	94.76	90.00	547.539
	1.52	6.950	5.493	14.147	90.00	94.76	90.00	538.218
	1.72	6.862	5.496	14.078	90.00	94.76	90.00	1355.29
	2.8	6.66	5.447	13.895	90.00	94.76	90.00	502.331
	4.5	6.488	5.368	13.644	90.00	94.76	90.00	473.549
	5.5	6.323	5.411	13.441	90.00	94.76	90.00	458.281
β	Exp. atm	7.306	5.5093	14.0380	95.14	97.23	85.36	556.886
	Cal. atm	7.541	5.662	14.245	95.14	97.23	85.36	599.465
	0.25	7.467	5.630	14.188	95.14	97.23	85.36	587.867
	0.45	7.421	5.600	14.146	95.14	97.23	85.36	579.411
	0.65	7.374	5.573	14.107	95.14	97.23	85.36	571.386
	1	7.310	5.534	14.052	95.14	97.23	85.36	560.270
	1.52	7.226	5.494	13.978	95.14	97.23	85.36	546.933
	1.72	7.198	5.479	13.953	95.14	97.23	85.36	542.355
	2.8	7.004	5.419	13.820	95.14	97.23	85.36	516.983
	4.5	6.757	5.403	13.641	95.14	97.23	85.36	490.837
	5.5	6.464	5.482	13.445	95.14	97.23	85.36	469.574
γ	Exp. atm	15.260	5.962	14.615	90	115.56	90	599.75
	Cal. atm	15.155	5.923	14.661	90	115.56	90	593.612
	0.25	15.117	5.888	14.619	90	115.56	90	586.938
	0.45	14.998	5.833	14.590	90	115.56	90	575.734
	1.52	14.787	5.714	14.390	90	115.56	90	548.432
	1.72	14.744	5.691	14.348	90	115.56	90	543.046
	2.8	14.475	5.639	14.172	90	115.56	90	521.787
